# Ambient temperature and kidney function in primary care patients

**DOI:** 10.1007/s40620-023-01715-8

**Published:** 2023-08-23

**Authors:** Simeon Schietzel, Stefan Zechmann, Fabio Valeri, Maria Staudinger, Pietro Cippà, Jan Seibert, Oliver Senn, Harald Seeger

**Affiliations:** 1 Divison of Nephrology, University Hospital Bern, Bern, Switzerland; 2 Institute of Primary Care, University of Zurich and University Hospital Zurich, Zurich, Switzerland; 3 Department of Geography, University Zurich, Zurich, Switzerland; 4 Division of Nephrology, Ente Ospedaliero Cantonale, Lugano, Switzerland; 5 Division of Nephrology, University Hospital Zurich, Rämistrasse 100, 8091 Zurich, Switzerland

**Keywords:** Temperature, Kidney, Injury, Climate, Weather, Dehydration

## Abstract

**Introduction:**

Exposure to high ambient temperatures is associated with a risk of acute kidney injury. However, evidence comes from emergency departments or extreme weather exposures. It is unclear whether temperature-related adverse kidney outcomes can also be detected at a community level in a temperate climate zone.

**Methods:**

In a 9.5-year retrospective cohort study we correlated estimated glomerular filtration rate (eGFR) values of Swiss adult primary care patients from the FIRE cohort (Family medicine Research using Electronic medical records) with same-day maximum local ambient temperature data. We investigated 5 temperature groups (< 15 °C, 15–19 °C, 20–24 °C, 25–29 °C and  ≥ 30 °C) as well as possible interactions for patients with increased kidney vulnerability (chronic heart failure, diabetes, chronic kidney disease, therapy with renin–angiotensin–aldosterone-system (RAAS) inhibitors, diuretics or non-steroidal anti-inflammatory drugs).

**Results:**

We included 18,000 primary care patients who altogether provided 132,176 creatinine measurements. In the unadjusted analysis, higher ambient temperatures were associated with lower eGFR across all age and vulnerability groups. In the adjusted models, we did not find a consistent association.The highest ambient temperature differences (> 25 or > 30 versus < 15 °C) were associated with marginally reduced kidney function only in patients with ≥ 3 risk factors for kidney vulnerability, with a maximum estimated glomerular filtration rate reduction of −2.9 ml/min/1.73m^2^ (SE 1.0), *P* 0.003.

**Discussion:**

In a large primary care cohort from a temperate climate zone, we did not find an association between ambient temperatures and kidney function. A marginal inverse association in highly vulnerable patients is of unclear clinical relevance.

**Graphical abstract:**

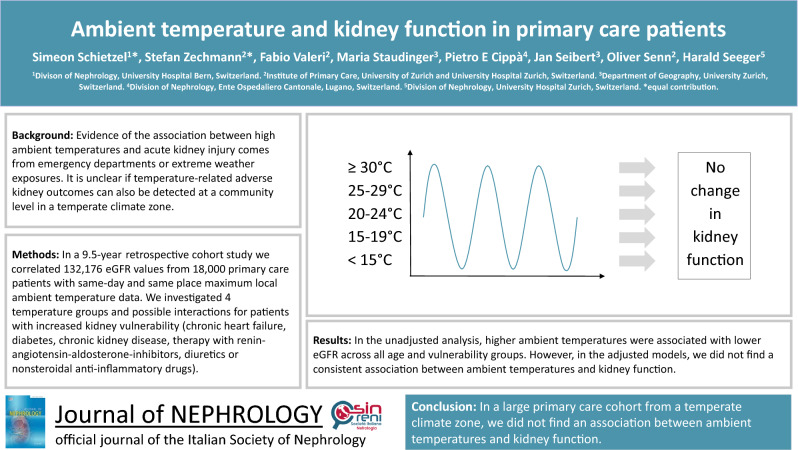

**Supplementary Information:**

The online version contains supplementary material available at 10.1007/s40620-023-01715-8.

## Introduction

Interest in heat-related kidney injury has considerably increased during recent years. Ninety-three percent of all research articles on the impact of heat on kidney health have been published within the last decade [1].

The fact that heat stress increases the risk of dehydration-related kidney damage is intuitive and beyond controversy [2]. Evidence includes meta-analytic data of increased hospitalizations with acute kidney injury (AKI) during periods of higher ambient temperatures [3] and investigations of acute and chronic kidney damage following occupational heat exposures from sunlight in agricultural and construction work or from heat processes in factories [4, 5].

However, evidence comes from emergency departments, hospital admissions or extreme scenarios like long strenuous work in very high temperatures. It is unclear whether temperature-related adverse kidney outcomes can also be detected in non-hospital-based general medicine practices within the community. This is of interrest especially in the context of global warming.

In view of the repeatedly established association between high ambient temperatures and emergency department visits with adverse kidney outcomes, we hypothesized that kidney function might be affected to some degree by temperature at the level of pre-hospital general medicine.

A Japanese study involving 102 hypertensive community-dwelling patients found reduced kidney function during summer in those with and without chronic kidney disease (CKD) [6]. Relative declines in estimated glomerular filtration rate (eGFR) were greater in CKD patients with an eGFR decrease of −13.8% (SD 9.4) during summer. However, a Chinese study of 109 community-dwelling CKD patients did not find such an association although blood pressure values were inversely correlated with outdoor temperatures [7]. Larger studies on seasonal temperature changes and kidney function in community settings do not exist. Kidney protective counseling has proven successful in very hot weather scenarios [8]. If we were to observe that kidney function is affected by ambient temperatures even within non-emergency settings, patient care could be improved by placing increased focus on patients at risk.

Taking advantage of both a large database, representative of the Swiss primary care population, and national meteorology data with highly accurate temporal and spatial resolution, we were able to match ambient temperatures at the general practitioner (GP) clinics with patients` creatinine values and medical data. We are the first to provide data on the association of ambient temperatures and kidney function in a large primary care cohort living in a temperate climate zone.

## Methods

This is a retrospective cohort study: we analyzed data of primary care patients registered in the FIRE (Family medicine—Research using Electronic medical records) database. The FIRE project was established in 2009 and is an ongoing research project at the Institute of Primary Care of the University of Zurich, Switzerland. It provides structured routine medical data from Swiss primary care [10–12]. In brief, the database covers patient demographics, vital signs, laboratory data and prescribed medication according to Anatomical Therapeutic Chemical (ATC) coding [13, 14] as well as diagnoses based on the ICPC-2 classification (International Classification of Primary Care 2) [15]. Participating GPs extract data in an anonymized way from their electronic medical records. These data are then centrally pooled in the FIRE database and aggregated by individual consultation dates. Until August 2018, more than 400 GPs participated, representing over 5 million consultations.

### Inclusion and exclusion criteria

We considered all patients in the FIRE database for whom creatinine measurements were available between 1st January, 2009 and 31st August, 2018 as potentially eligible. We excluded patients < 18 and > 100 years of age and those with < 4 creatinine measurements. We used the highest eGFR value of each patient to define his or her baseline kidney function. Therefore, we excluded these values from the analysis. A flowchart of patients and data selection process is presented in Supplemental Fig. 1.

### Definitions

#### Kidney function

We calculated creatinine based eGFRs according to the chronic kidney disease epidemiology collaboration (CKD—EPI) formula [16] to describe the level of kidney function on a given day. For the definition of kidney disease as a vulnerability factor, we considered an individual as being affected by CKD when his/her highest eGFR value (calculated from the lowest creatinine measurement) was < 60 ml/min/1.73m^2^.

#### Temperature

To determine the temperature that an individual patient was exposed to on a given day of creatinine measurement, we synchronized the SwissMeteo-dataset (stratified by region) with geographical information of the individual patient’s GP’s practice. We used the maximum temperature of the day during which eGFR was measured. For the analyses we stratified the temperature into 5 ranges, < 15 °C, 15–19 °C, 20–24 °C, 25–29 °C and ≥ 30 °C. The MeteoSwiss-dataset contains maximum daily temperatures as gridded data with a spatial resolution of 2.2 km. These data were interpolated based on measured air temperatures at 2 m above ground level from approximately 90 stations and considering elevations [17]. The uncertainty of the gridded temperature values depends on the network density and hence varies from location to location. The uncertainty is of about 0.6 °C, but slightly higher during winter. A detailed discussion on the interpolation method and the associated uncertainties is provided by Frei (2014). For each GP, the temperature was interpolated from the neighboring cells with a weight given to the distance from the GP to the center of the grid cell.

#### Kidney vulnerability factors

We defined five factors that potentially increase kidney vulnerability to high ambient temperature with respect to kidney function: heart failure (HF), CKD, diabetes mellitus, RAAS inhibitors or diuretics and non-steroidal anti-inflammatory/antirheumatic drugs.

Applying these factors, we stratified our cohort into individuals with no kidney vulnerability factor (= vulnerability group 0) and those with 1, 2, 3, 4 or 5 vulnerability factors (vulnerability groups 1–5). Group 1 includes patients who exhibit any one of the above-mentioned vulnerability factors (no matter which one), while group 5 patients exhibit all 5 of the above-mentioned vulnerability factors. For the definition of the vulnerability factors and chronic diseases, we used the International Classification of Primary Care version 2 (ICPC-2) [15] adapted by O’Halloran et al. [14], the Anatomical Therapeutic Chemical (ATC) codes of medication adapted by Lamers et al. [18] as well as clinical measurements from the primary care visits. We defined HF by ICPC code K77 and diabetes mellitus using ICPC codes T89, T90, ATC code A10 as well as HbA1c values > 6.5%. We defined CKD as a creatinine-based eGFR of < 60 ml/min/1.73m^2^ according to CKD–EPI. We defined RAAS inhibitors by ATC code C09, diuretics by ATC codes C03 and non-steroidal anti-inflammatory/antirheumatic drugs by ATC codes M01A and M01B. We included only these medications, prescribed at least twice and at least six months apart, thus ensuring the inclusion of long-term medication.

#### Other patient characteristics

We used the following patient characteristics to further describe our cohort: age (stratified as ≤ 39, 40–49, 50–59, 60–69, 70–79, ≥ 80 years of age), body mass index (BMI) (stratified according to WHO) [19], arterial hypertension (presence and grading based on the mean value of all blood pressure measurements of each individual during the study, stratified according to European Society of Cardiology/European Society of Hypertension (ESC/ESH 2018) [20], CKD eGFR categories (according to KDIGO 2012) and number of chronic diseases (according to the International Classification of Primary Care version 2 (ICPC-2) [15] adapted by O’Halloran et al. [14], ATC codes of medication adapted by Lamers et al. [18] as well as clinical measurements from the primary care visits.

### Statistical analysis

Descriptive data are presented as frequencies and percentages, mean and standard deviation (SD), or median and interquartile range (IQR), where appropriate. If not indicated otherwise, all percentages refer to the total number of included patients. For the univariate tables we used all available creatinine measurements of the included patients (dataset A). To visually present the effect of temperature on eGFR, a plot was created considering age group and vulnerability. Since the huge amount of single data points would not provide any information, a smoothed line was added using cubic spline and a 95% confidence region. To consider maximal eGFR of the patient as an explainable variable in the regression analysis, the maximum eGFR observation was removed from dataset A resulting in a reduction of creatinine measurements available for further analysis (dataset B). To study the effect of each variable of interest we performed a 3-level (measurment, patient and practice), bivariable and multivariable regression, also known as a mixed-model. Bivariable regression contains age and one other variable. Age was centered with a median age of 71. To consider the nonlinearity of age we used a cubic B-splines smoothing technique with 15 knots. We included interaction effect between temperature category and vulnerability level. Temperature was categorized: < 15, 15–19, 20–24, 25–29, ≥ 30 °C. To avoid data loss due to missing BMI values, patients without BMI value were categorized as not available (NA). Taken together, the multivariable regression is: GFR = B–Splines of age (centered) + sex + BMI-group + hypertension category + number of chronic disease + eGFR category according to KDIGO 2012 + temperature category x vulnerability + random effect of practice + random effect of patient within practice. Data were analyzed using R^®^ Version 3.5.0.

## Results

We included 18,000 patients from 51 GP practices who provided a total of 132,176 creatinine measurements. Mean age was 68.8 (SD 14.8) years, 51.8% were female. 31.4% suffered from chronic kidney disease, 24% had diabetes, mean BMI was 28.0 kg/m^2^ (SD 5.6) and 46.4% showed at least first grade mean hypertensive blood pressure values during the study period. Mean number of chronic diseases was 3.9 (SD 3.0) and patients received a cumulative average of 8.7 (SD 7.3) medications during the study period. Based on the highest eGFR value of each participant throughout the study period, patients were subdivided according to KDIGO 2012 as follows; 51.3% as G1, 38.1% as G2, 6.8 and 3% as G3a and 3b, respectively, 0.7% as G4, 0.1% as G5. Mean eGFR was 72.6 ml/min/1.73m^2^ (SD 25.5) in men and 75.5 ml/min/1.73m^2^ (SD 24.7) in women (Table 1).

**Table 1:** Characteristics of study participants

Total	No. of patients (%)		No. of measurements (%)		GFR mean (SD)	
	18,000	(100%)	132,176	(100%)	74.0	(25.2)
Age class						
< 40	1153	(4.5)	5548	(4.2)	108.5	(23.4)
40–49	2244	(8.7)	9295	(7.0)	97.8	(19.3)
50–59	4157	(16.1)	18,699	(14.1)	89.8	(18.9)
60–69	5852	(22.7)	27,181	(20.6)	79.7	(19.5)
70–79	6624	(25.7)	36,317	(27.5)	68.4	(20.2)
80–89	4719	(18.3)	29,446	(22.3)	56.7	(20.4)
≥ 90	1039	(4.0)	5690	(4.3)	47.7	(18.1)
Gender						
Female	9315	(51.8)	68,182	(51.6)	72.6	(25.5)
Male	8685	(48.3)	63,994	(48.4)	75.5	(24.7)
BMI (kg/m^2^)						
< 18.5	422	(1.5)	1700	(1.3)	78.2	(28.4)
18.5–24.9	5417	(19.8)	28,181	(21.3)	75.2	(24.2)
25.0–29.9	7117	(26.1)	36,526	(27.6)	73.6	(23.7)
30.0–34.9	4011	(14.7)	19,339	(14.6)	74.6	(24.6)
35.0–39.9	1536	(5.6)	7367	(5.6)	75.1	(26.0)
> 40	549	(2.0)	2851	(2.2)	73.3	(27.8)
NA	8248	(30.2)	36,212	(27.4)	72.9	(27.0)
Blood pressure control during study period^a^						
Normotensive	8999	(50.0)	67,552	(51.1)	74.7	(25.8)
Grade 1	6276	(34.9)	46,343	(35.1)	72.7	(23.9)
Grade 2	1629	(9.1)	11,195	(8.5)	75.0	(23.4)
Grade 3	427	(2.4)	2818	(2.1)	76.0	(25.1)
NA	669	(3.7)	4268	(3.2)	73.5	(32.2)
Chronic kidney disease						
No	16,093	(68.6)	93,559	(70.8)	86.9	(16.2)
Yes	7382	(31.4)	38,617	(29.2)	42.8	(12.4)
Diabetes						
No	13,638	(75.8)	95,396	(72.2)	75.4	(24.8)
Yes	4362	(24.2)	36,780	(27.8)	70.3	(25.8)
RAAS Inhibitors/Diuretics						
No	6317	(35.1)	38,919	(29.4)	85.9	(22.6)
Yes	11,683	(64.9)	93,257	(70.6)	69.1	(24.5)
Non-steroidal anti-inflammatory drugs						
No	7830	(43.5)	57,057	(43.2)	69.1	(26.4)
Yes	10,170	(56.5)	75,119	(56.8)	77.8	(23.5)
Chronic heart failure (HF)						
No	7153	(95.3)	122,396	(92.6)	75.6	(24.7)
Yes	847	(4.7)	9780	(7.4)	54.7	(23.0)
Number of chronic diseases						
0	4386	(11.6)	12,273	(9.3)	78.6	(26.4)
1	6304	(16.7)	17,632	(13.3)	82.2	(24.0)
2	6988	(18.5)	20,143	(15.2)	78.5	(24.5)
3	6725	(17.8)	19,881	(15.0)	75.4	(24.5)
4	5673	(15.1)	17,040	(12.9)	73.4	(24.7)
5–10	6845	(18.2)	40,361	(30.5)	68.6	(24.0)
≥ 11	771	(2.0)	4846	(3.7)	56.6	(23.9)
eGFR category						
> 90 ml/min/1.73m^2^	9241	(51.3)	64,391	(48.7)	91.5	(18.2)
60–89 ml/min/1.73m^2^	6852	(38.1)	51,037	(38.6)	64.5	(15.1)
45–59 ml/min/1.73m^2^	1223	(6.8)	10,822	(8.2)	41.0	(10.4)
30–44 ml/min/1.73m^2^	536	(3.0)	4492	(3.4)	30.1	(7.9)
15–29 ml/min/1.73m^2^	134	(0.7)	1322	(1.0)	18.1	(5.4)
< 15 ml/min/1.73m^2^	14	(0.1)	112	(0.1)	17.4	(5.8)
Kidney vulnerability groups						
0	2088	(8.9)	10,912	(8.3)	90.4	(18.1)
1	6848	(29.2)	36,894	(27.9)	85.9	(19.6)
2	8298	(35.3)	46,655	(35.3)	73.9	(23.8)
3	4893	(20.8)	28,497	(21.6)	62.0	(24.6)
4	1240	(5.3)	8232	(6.2)	45.6	(16.8)
5	108	(0.5)	986	(0.7)	40.0	(11.6)
Temperature category						
< 15 °C	17,450	(31.9)	67,754	(51.3)	74.6	(25.0)
15–20 °C	12,793	(23.4)	24,729	(18.7)	74.5	(25.0)
20–25 °C	12,000	(21.9)	21,926	(16.6)	73.3	(25.3)
25–30 °C	8837	(16.2)	13,482	(10.2)	72.3	(25.7)
≥ 30 °C	3627	(6.6)	4285	(3.2)	71.8	(25.7)

Patient and measurement distribution of categorical variables and eGFR mean plus standard deviation (SD) for each category. *NA* not available. The sum of patients for a category may be larger than 18,000 since patients may change levels within the category over time. Based on dataset A (*n* = 132,176 176). ^a^based on mean values of all blood pressure measurements of each individual during the study

### eGFR, age and temperature

To highlight how age affected eGFR, all GFR estimates were plotted against age. From age 40 onward, eGFR decreased by 10 mL/min1.73m^2^ for each 10 year increase (Supplemental Fig. 2).

### Temperature

**Fig. 1:**
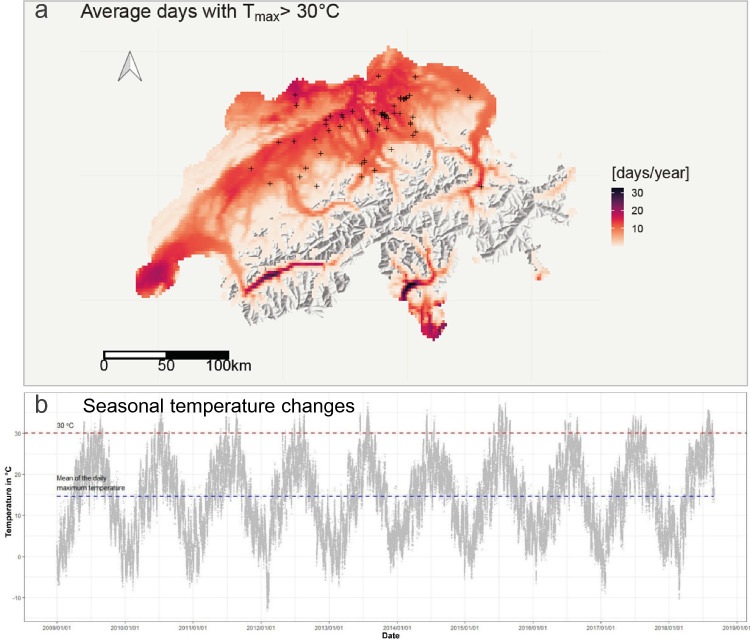
Spatial distribution and frequency of temperatures > 30 °C (**a**) and temperature changes (**b**) during the course of the study. **a** The map shows the average number of days per year during which the threshold temperature of 30 °C was exceeded throughout the study period. The black crosses indicate the location of the GPs. Grid cells that never exceeded the threshold are not shown. The source of the underlying relief map is the Swiss Federal Office of Topography. **b** Each point is the maximum of the temperature for each GP practice on each day. *GP* general practitioner. Dashed blue line represents mean of the daily maximum temperature over the entire observation period. Dashed red line represents temperature threshold of 30 °C

For the 132,176 creatinine measurements at the GP clinics, we had corresponding temperature data (same day, same area) for 100% of the cases. During the 9-year study period, mean maximum daily temperature was 14.6 °C (SD 9.0). Seasonal temperature changes are shown in Fig. 1b. During winter (December 22nd to March 20th), mean maximum daily temperature was 5.8 °C (SD 5.3), while it was 25.7 °C (SD 5.0) during summer (June 21st–September 23rd). Temperature ranged from a minimum of −13.4 °C to a maximum of 37.4 °C. We identified 5743 patients and 7474 measurements meeting temperature ≥ 30 °C. The average number of days per year during which the temperature exceeded 30 °C for the study period varied from zero to 33 days (Fig. 1a).

### eGFR according to ambient temperature in the entire cohort

**Fig. 2:**
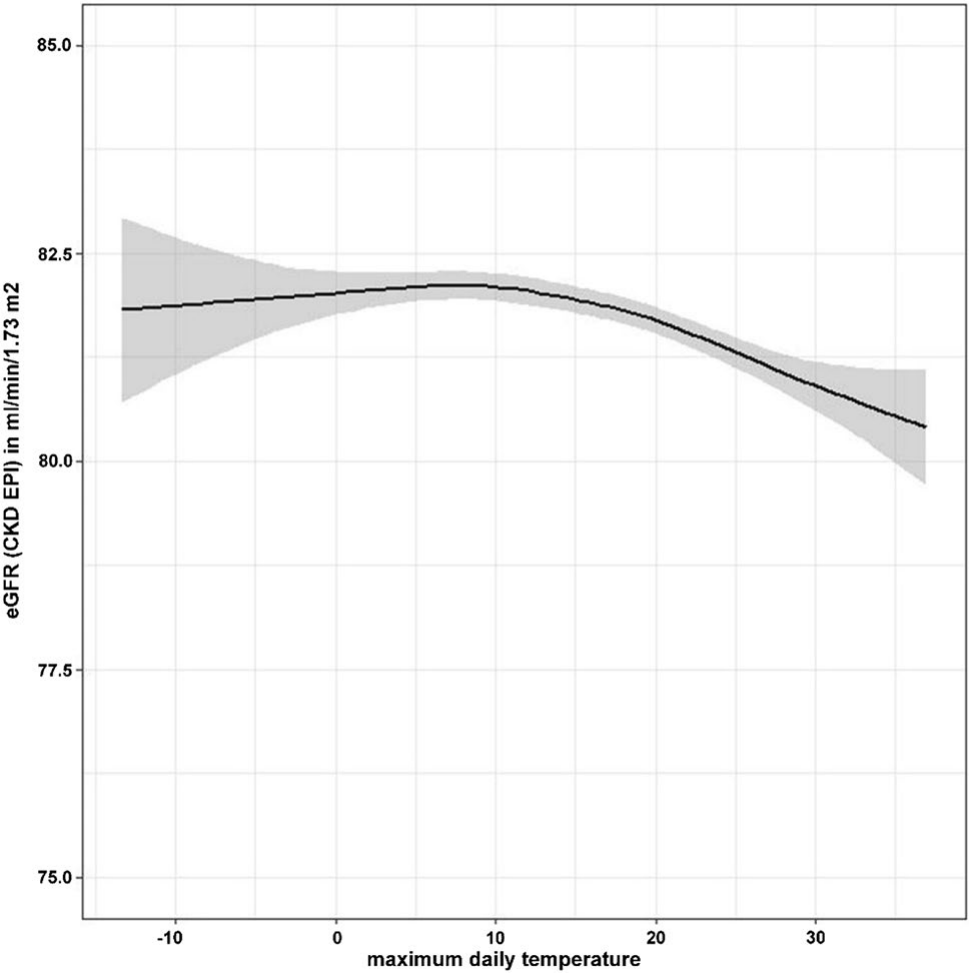
Unadjusted association between ambient temperature and kidney function. Unadjusted association between ambient temperature and eGFR using the full range of temperature values and all 132,176 creatinine values from the 9.5-year observation period. *eGFR* estimated glomerular filtration rate

In the overall population, the unadjusted association of ambient temperature with eGFR was significant but discrete as can be appreciated in Fig. 2. Starting at ambient temperatures of around 10 °C, the higher the maximum daily temperature, the lower the associated eGFR value (Fig. 2).

#### Kidney vulnerability

In our population, 2088 (8.9%) participants had no vulnerability condition (group 0). Among participants, 6848 (29.2%), 8298 (35.3%), 4893 (20.8%), and 1240 (5.3%) had one, two, three and four vulnerability conditions (group 1 to 4), respectively, and 108 (0.5%) participants exhibited all five vulnerability conditions (group 5). The sum of all patients is higher than the number of included patients since any given patient could change vulnerability status during the study period. Supplemental Fig. 3 shows the distribution of the vulnerability conditions according to vulnerability group.

### Unadjusted analysis of kidney vulnerability subgroups

**Fig. 3:**
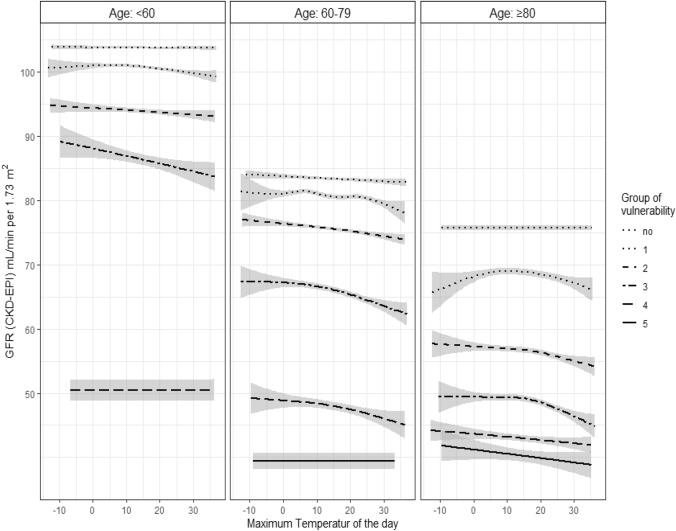
Unadjusted association between temperature and kidney function according to age and number of vulnerabilities. Smoothed curve of eGFR values against temperature with 95% confidence region considering age and vulnerability. Since age group < 60 with vulnerability = 5 only entailed 4 patients with 21 observations it was not considered in the plot. *eGFR* estimated glomerular filtration rate

The greater the presence of kidney vulnerability conditions, the lower the observed eGFR. This was expected since CKD was one of the vulnerability conditions and the other risk factors such as HF, diabetes mellitus or chronic NSAID use can all cause a chronic decrease in eGFR. In addition, RAAS inhibitor treatment leads to a reduction in eGFR by reducing glomerular filtration. Figure 3 displays the smoothed curve of eGFR values against temperature with 95% confidence interval considering age group and vulnerability. Confidence intervals widen at both ends of the curves due to a lower number of data points at extremes of temperatures. Interestingly, in patients without kidney risk factors there was no association between temperature and eGFR regardless of age. The presence of two or more vulnerability conditions however, seems to increase the risk of eGFR deterioration with higher temperatures, again regardless of age.

### Multivariable analysis regarding the influence of vulnerability status on temperature sensitivity of eGFR

As was seen in the unadjusted analysis, in the multivariable model we observed a significant negative correlation between the number of vulnerability factors and eGFR at T < 15 °C, (Table 2). In patients with zero kidney vulnerability factors we did not find an independent association between ambient temperature and eGFR except for the comparison between the temperature groups of 25–30 °C versus < 15 °C with an eGFR decline of −0.7 ml/min/1.73m^2^ (SE 0.3); *P* 0.03 **(**Table 2**)**. With regard to the subgroups of patients exhibiting conditions that increase kidney vulnerability, significant inverse associations with eGFR were seen in vulnerability groups 3, 4 and 5 at higher temperatures. In group 3, the effect was −1.5 mL/min/1.73m^2^ at ≥ 30 °C (SE 0.6); *P* 0.015, in group 4 it was −1.9 mL/min/1.73m^2^ at ≥ 30 °C (SE 0.7); *P* 0.01, and in group 5 it was −2.9 mL/min/1.73m^2^ at 25–29 °C (SE 1.0); *P* 0.003. The effect at ≥ 30 °C was not significant; however there was only a low number of data points. In the remaining temperature and vulnerability groups, we did not detect a significant association between ambient temperature and eGFR, (Table 2). Taken together, at least > two risk factors had to be present to detect a robust association between higher outdoor temperature and lower eGFR. Except for gender, all factors chosen for adjustment were independently associated with significant eGFR changes (Supplemental Table 1).

**Table 2:** Multivariate regression analysis associating quintiles of ambient temperature with eGFR in different vulnerability groups

	No. of measurements	Bivariable regression		Multivariable regression	
		Coefficient = Change in eGFR in ml/min (SE)	*P* Value	Coefficient = Change in eGFR in ml/min (SE)	*P* Value
Vulnerability (at T < 15 °C)					
0	5658	0.00			
1	18,931	−8.46 (0.27)	0.000	−5.4 (0.3)	0.000
2	24,048	−21.42 (0.29)	0.000	−15.6 (0.3)	0.000
3	14,396	−37.54 (0.32)	0.000	−29.4 (0.3)	0.000
4	4220	−56.29 (0.37)	0.000	−46.4 (0.4)	0.000
5	501	−77.12 (0.68)	0.000	−65.1 (0.7)	0.000
Vulnerability 0					
< 15 °C	5658	0.00		0.0	
15–20 °C	2054	−0.38 (0.08)	0.000	−0.2 (0.3)	0.359
20–25 °C	1744	−0.71 (0.08)	0.000	−0.4 (0.3)	0.116
25–30 °C	1099	−0.70 (0.10)	0.000	−**0.7 (0.3)**	**0.030**
≥ 30 °C	357	−1.01 (0.16)	0.000	0.0 (0.53)	0.958
Vulnerability 1					
15–20 °C	6963			−0.2 (0.3)	0.424
20–25 °C	6106			−0.2 (0.3)	0.602
25–30 °C	3716			0.3 (0.4)	0.445
≥ 30 °C	1178			−1.0 (0.6)	0.106
Vulnerability 2					
15–20 °C	8719			0.0 (0.3)	0.979
20–25 °C	7642			−0.1 (0.3)	0.793
25–30 °C	4737			0.0 (0.4)	0.989
≥ 30 °C	1509			−0.7 (0.6)	0.224
Vulnerability 3					
15–20 °C	5295			0.0 (0.3)	0.995
20–25 °C	4874			−0.3 (0.3)	0.306
25–30 °C	2984			−0.2 (0.4)	0.678
≥ 30 °C	948			−**1.5 (0.6)**	**0.015**
Vulnerability 4					
15–20 °C	1500			−0.4 (0.4)	0.244
20–25 °C	1396			−0.4 (0.4)	0.269
25–30 °C	855			−0.4 (0.4)	0.374
≥ 30 °C	261			−**1.9 (0.7)**	**0.010**
Vulnerability 5					
15–20 °C	501			0.8 (0.7)	0.287
20–25 °C	198			0.4 (0.8)	0.629
25–30 °C	164			−**2.9 (1.0)**	**0.003**
≥ 30 °C	91			−1.4 (1.5)	0.365

Analysis based on dataset B (*n* = 114 176)

Changes in eGFR values according to temperature groups that reached significance in the multivariable regression and corresponding P values are marked in bold

## Discussion

The main result  of our study is that in a population encompassing 18,000 Swiss primary care patients, ambient temperatures > 25 °C (compared to < 15 °C) were significantly associated with a mild eGFR decline (−2.9 ml/min/1.73m^2^) only in patients with more than two kidney vulnerability factors.

Other studies investigating community settings were smaller or focused on special disease populations. Older hypertensive Japanese adults with and without CKD showed decreased eGFR values during summer (eGFR-7.1 ± 7.0 and −5.8 ± 5.2 ml/min/1.73m^2^, respectively) [6]. Italian elective cardiac surgery patients displayed elevated creatinine values during episodes of increased ambient temperatures (103 ± 61.6 vs. 98.7 ± 74.8 μmol/L, *P* < 0.001) [9]. However, Chinese CKD patients showed no association of kidney function with ambient temperature [7].

A recent meta-analysis suggested a relative risk (RR) of 1.012 (95% CI 1.009–1.015) for acute kidney injury (AKI) for every 1 °C increase in ambient temperature [3]. However, studies were exclusively conducted in hospital or emergency department settings. We might have missed more severe health conditions such as AKI during high ambient temperatures as these patients may have presented directly to the hospital and not to their GP.

Several factors potentially explain the absence of a robust correlation between ambient temperatures and kidney function in patients without kidney vulnerability. First, temperatures in our temperate climate zone might not be extreme enough to overwhelm the body’s compensatory mechanisms. Second, we assessed neither the participant’s behaviors and location, nor individual body temperature. Hence, it is unknown which temperatures individuals were actually exposed to. We used maximum temperature on the day of creatinine measurement, with the hypothesis of maximum volume depletion risk at the highest temperatures. Others used daytime or an average of temperatures over several days [22, 23], potentially highlighting more sustained and therefore more pronounced dehydration stressors. However, the extent of daily temperature variability is not captured by both approaches and comparative studies are needed.

With regard to kidney vulnerability, Xu et al. also found an increased risk of worsening kidney function during high ambient temperatures in patients with HF, CKD, diabetes and hypertension, which validates the conditions we chose [24]. Interestingly, our analysis of kidney vulnerability did not show age as a relevant risk factor for temperature-associated eGFR decrease.

The temperature-associated changes of eGFR we found in highly vulnerable patients may appear to be clinically negligible. However, annual eGFR changes of comparable magnitude have been associated with significantly increased mortality [25, 26]. Furthermore, as kidney autoregulation usually maintains GFR independent of systemic blood pressure [27], a mild decrease in eGFR may indicate relevant hemodynamic alterations, which potentially increase the risks for other circulatory sequelae such as stroke or myocardial ischemia.

Our study has several strengths. It is the first investigation of ambient temperature and kidney function in a large, unselected primary care cohort, providing more than 9 years of real life data for a western European population in tempered continental weather conditions. Analyzed data are of high validity as they are extracted from digital records ascertained and documented exclusively by GPs. Our study also has limitations. We could not ascertain patient behaviors or body temperatures, hence, we do not know which temperature an individual patient was exposed to on a given day. Further, a creatinine value measured during high temperatures was not necessarily from the same patient as a creatinine value measured during low temperatures in our comparative analysis. However, as our cohort comprises 132,176 creatinine measurements from 18,000 patients and as we adjusted for important confounders, we consider our results to be valid even without data on the individual’s longitudinal follow-up.

In summary, we did not find a biologically significant association between seasonal temperature variability and kidney function within a general medicine ambulatory care population. This is somewhat in contrast with data from emergency and hospital settings, where an association between ambient temperatures and kidney damage has repeatedly been observed. With regard to potential public health implications, and in light of global warming, further research is needed to understand how data from general medicine practices may contribute to identifying individual patients at risk so as to prevent temperature-related kidney damage. For future studies, prospective designs and both incorporation of behavioral data and individuals’ body temperatures might provide a considerable contribution to the topic.

### Supplementary Information

Below is the link to the electronic supplementary material.

Supplementary file1 (PDF 508 KB)

## Data Availability

We will share the data of our investigation upon reasonable request.

## Declarations

### Patient and public involvement

With regard to our investigation, we did not directly involve patients or the public in the planning, execution and reporting of the research. However, general practitioners participating in FIRE are informed and updated on a regular basis regarding ongoing projects and published results and can contribute to new projects. Detailed information about the FIRE database, its scientific and clinical purpose and all publications are presented on the web https://www.hausarztmedizin.uzh.ch/de/fire2.html.

### Dissemination to participants and related patient and public communities

Published results will be made available on the FIRE website https://www.hausarztmedizin.uzh.ch/de/fire2.html. Participating general practitioners will be informed about the results during the subsequent rotational FIRE meeting.
