# Supplementary material for: Ambient temperature and kidney function in primary care patients
Source: J Nephrol. 2023 Aug 23;37(1):95–105. doi: 10.1007/s40620-023-01715-8 (PMC10920449; doi:10.1007/s40620-023-01715-8)
Supplement: Supplementary file 1 — Supplementary file1 (PDF 508 KB) [file 40620_2023_1715_MOESM1_ESM.pdf]

Supplemental Material

Supplemental Table 1. Associations of our adjustment factors with the outcome eGFR.

	Bivariable regression			Multivariable regression		
	Coefficient	Standard error	P-Value	Coefficient	Standard error	P-Value
**Intercept**				121.9	1.10	0.000
**Sex (male vs female)**	0.48	0.23	0.042	0.03	0.2	0.132
**Age**						
degree=3, knots=15:1				-23.7	1.6	0.000
degree=3, knots=15:2				-20.9	0.8	0.000
degree=3, knots=15:3				-42.1	1.1	0.000
degree=3, knots=15:4				-47.6	1.5	0.000
**BMI group**						
Normal	0.00					
Low	0.40	0.41	0.326	-0.18	0.4	0.602
Overweight	-0.53	0.15	0.000	0.47	0.1	0.000
Grade 1	-1.10	0.19	0.000	1.05	0.2	0.000
Grade 2	-1.10	0.26	0.000	1.91	0.2	0.000
Grade 3	-2.35	0.41	0.000	1.61	0.4	0.000
NA	-2.18	0.16	0.000	0.43	0.1	0.003
**Hypertension group**						
No grade	0					
Grade 1	0.73	0.26	0.005	1.8	0.2	0.000
Grade 2	0.67	0.42	0.112	1.8	0.4	0.000
Grade 3	-1.16	0.77	0.132	1.2	0.6	0.055
**Number of chronic diseases**						
no	0.00					
1	1.99	0.18	0.000	1.7	0.2	0.000
2	2.56	0.19	0.000	2.2	0.2	0.000
3	2.92	0.20	0.000	2.5	0.2	0.000
4	2.96	0.21	0.000	2.5	0.2	0.000
5-10	2.71	0.21	0.000	2.0	0.2	0.000
≥ 11	0.77	0.31	0.014	0.4	0.3	0.114

**eGFR category according to KDIGO 2012**						
Stage 1	0.00					
Stage 2	-16.84	0.21	0.000	-13.4	0.3	0.000
Stage 3a	-36.61	0.37	0.000	-24.5	0.4	0.000
Stage 3b	-47.04	0.52	0.000	-36.0	0.6	0.000
Stage 4	-59.73	0.96	0.000	-49.2	1.2	0.000
Stage 5	-72.07	2.94	0.000	-67.4	4.0	0.000

Except for gender, all factors chosen for adjustment were independently associated with significant eGFR changes.

Supplemental Figure 1. Flowchart of patients and data selection process.

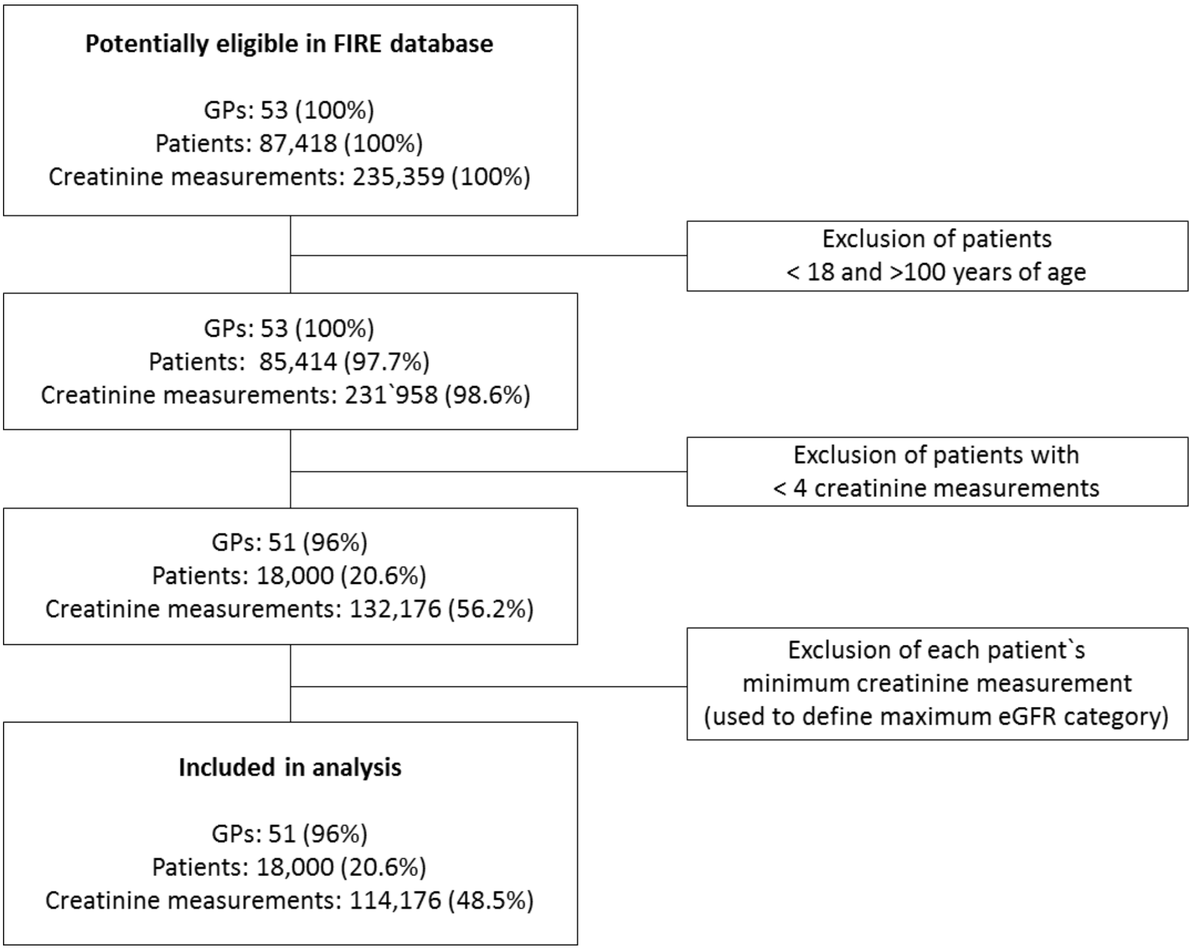

Flow chart shows total numbers (%) from initial screening to final inclusion for analysis. FIRE = family medicine ICPC research using electronic medical records, GP = general practitioner, eGFR = estimated glomerular filtration rate.

Supplemental Figure 2. Association between age and eGFR in our primary care cohort.

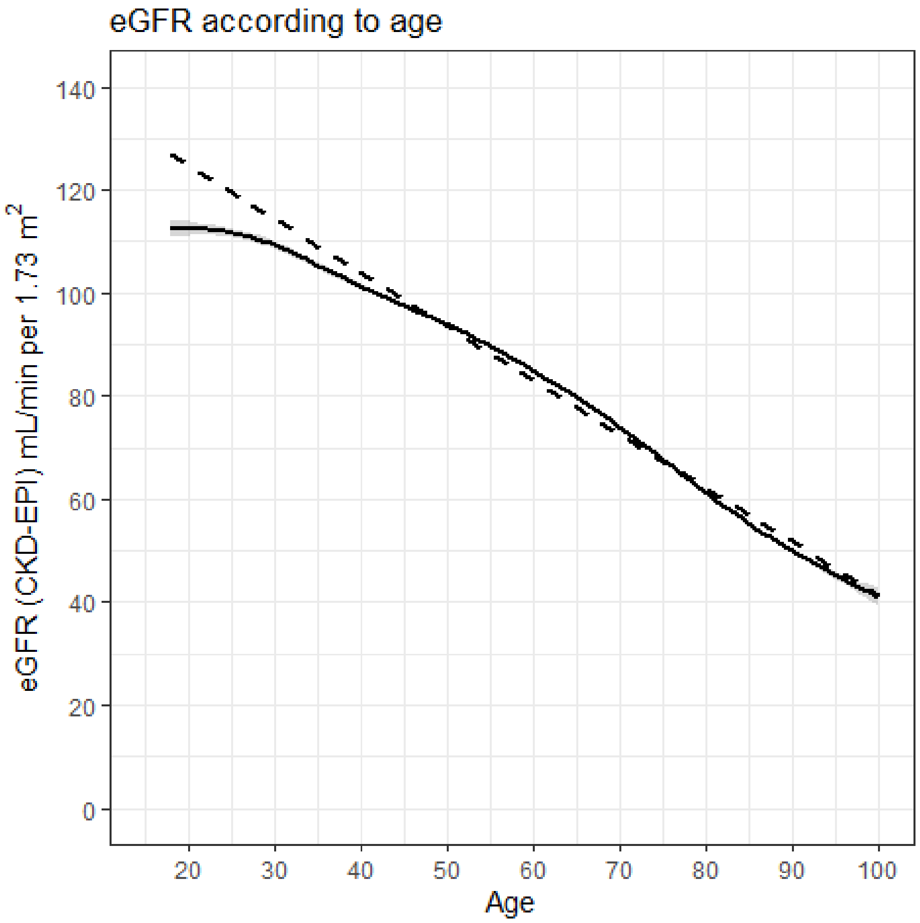

Solid line is a smoothed curve, dashed is a linear fit.

Supplemental Figure 3. Distribution of vulnerability conditions.

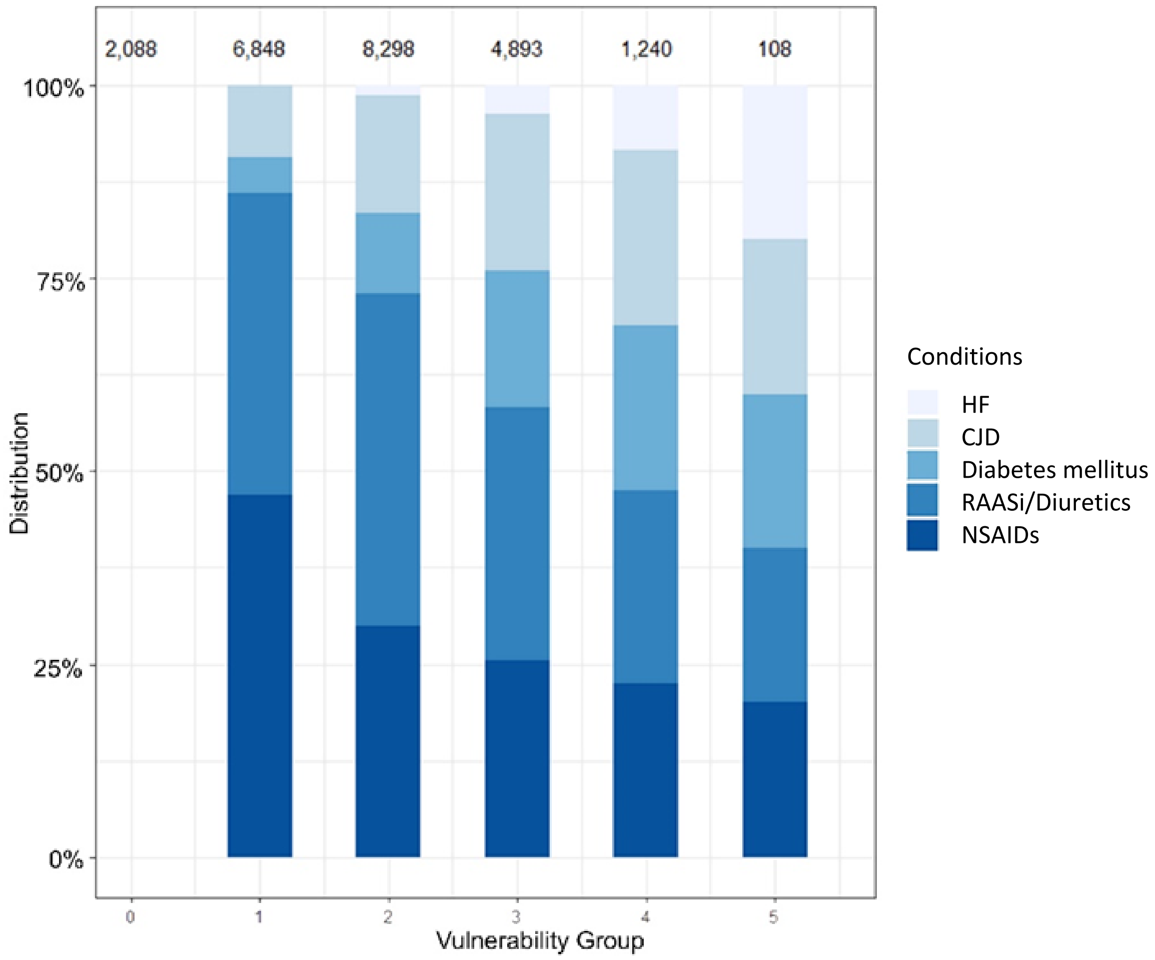

The numbers above the bars are the number of patients. In vulnerability group 1 each of the 6,848 patients has one of the 5 conditions. In group 2 each of the 8,298 patients has two of the 5 conditions etc. All of the 108 patients in group 5 have all five conditions. Abbreviations: HF = Heart failure, CKD = chronic kidney disease, RAASi/Diuretics = Renin-Angiotensin-Aldosterone-System inhibitors or diuretics, NSAIDs = non-steroidal anti-inflammatory/ antirheumatic drugs.
